# Increased risk of squamous cell carcinoma based on solar ultraviolet radiation measurements from outdoor workers in Lisbon, Portugal

**DOI:** 10.3389/fpubh.2026.1662734

**Published:** 2026-01-26

**Authors:** Marília Silva Paulo, Maria Miguel Castela, Claudine Strehl, Fernanda Carvalho, Tom Loney, Alberto Modenese, Fabriziomaria Gobba, Jorge Barroso-Dias, Cristina Pinho, Ana Rodrigues, Thomas Tenkate, Swen Malte John, Cara Bieck, Luís V. Lapão, Mélanie R. Maia, Stephan Westerhausen, Marc Wittlich

**Affiliations:** 1 CHRC, NOVA Medical School, Faculdade de Ciências Médicas, NMS, FCM, Universidade NOVA de Lisboa, Lisboa, Portugal; 2 NOVA National School of Public Health, Public Health Research Center, Comprehensive Health Research Center, CHRC, REAL, CCAL, NOVA University Lisbon, Lisbon, Portugal; 3 NOVA National School of Public Health, NOVA University Lisbon, Lisbon, Portugal; 4 Institute for Occupational Safety and Health of the German Social Accident Insurance (IFA), Sankt Augustin, Germany; 5 Portuguese Institute for Sea and Atmosphere, Lisbon, Portugal; 6 Center for Sci-Tech Research in Earth System and Energy - CREATE, IIFA, University of Évora, Évora, Portugal; 7 College of Medicine, Mohammed Bin Rashid University of Medicine and Health Sciences, Dubai Health, Dubai, United Arab Emirates; 8 Department of Biomedical, Metabolic and Neural Sciences, University of Modena and Reggio Emilia, Modena, Italy; 9 Departamento de Saúde, Higiene e Segurança, Câmara Municipal de Lisboa, Lisboa, Portugal; 10 Portuguese Society of Occupational Medicine, Working Committee “Work at Open Air”, Lisboa, Portugal; 11 School of Occupational and Public Health, Toronto Metropolitan University, Toronto, ON, Canada; 12 Department of Dermatology, Environmental Medicine, Health Theory, Osnabrück University, Osnabrück, Germany; 13 Institute for Interdisciplinary Dermatological Prevention and Rehabilitation (iDerm) at Osnabrück University, Osnabrück, Germany; 14 Lower-Saxonian Institute of Occupational Dermatology (NIB), Osnabrück, Germany; 15 Department of Occupational Medicine, Hazardous Substances and Health Sciences, Statutory Accident Insurance for the Health and Welfare Services, Hamburg, Germany; 16 Intelligent Decision Support Systems Laboratory, Research and Development Unit for Mechanical and Industrial Engineering (UNIDEMI), NOVA School of Science and Technology, Universidade NOVA de Lisboa, Lisbon, Portugal; 17 Laboratório Associado de Sistemas Inteligentes (LASI), Escola de Engenharia, Universidade do Minho, Guimarães, Portugal; 18 WHO Collaborating Centre on Health Workforce Policy and Planning, Global Health and Tropical Medicine, Universidade Nova de Lisboa, Lisbon, Portugal

**Keywords:** epidemiology of occupational exposures, occupational risks, outdoor workers, solar ultraviolet radiation, squamous cell carcinoma

## Abstract

**Introduction:**

Solar ultraviolet radiation (UVR) is one of the main causes of skin cancer, with squamous cell carcinoma (SCC) being particularly prevalent among outdoor workers due to chronic UVR exposure. Despite the increasing incidence of SCC in this group, cases remain under-reported and are not always classified as an occupational disease. Current guidelines for UVR exposure are established for a limit of 30 J/m^2^ over an 8-h workday, and they are implemented for both solar and artificial UVR (non-specific). This study aimed to calculate the excess risk of SCC among gardeners, gravediggers, pavers, asphalters, sanitation workers, and sailors in Lisbon based on measured solar UVR, in comparison with indoor workers.

**Methods:**

A prospective observational study using personal dosimeters was conducted to assess solar UVR in 90 outdoor workers from Lisbon Municipality, from April to October 2023. This data was used to calculate the relative risk (RR) of SCC for each of the investigated occupations as well as for each individual using a formula developed by Milon et al.

**Results:**

Solar UVR exposure was associated with an increased risk of developing SCC by values ranging from 22 to 437%, in terms of individual UVR dose assessment. Pavers had an increased risk of developing SCC by 65%, Asphalters by 133%, Sanitation Workers by 179%, Gravediggers by 187%, and Gardeners by 193%. Despite some limitations, a novel approach was tested by using direct UVR dose measurements in real environmental exposure conditions to estimate the risk of developing SCC.

**Discussion:**

Outdoor work is associated with a substantially increased risk of SCC. However, the current model needs to be refined to improve the accuracy of risk assessment and to support the development of targeted prevention interventions. The relevance of the study provides valuable insights for health and safety policies in reducing UVR exposure and SCC risk among outdoor workers.

## 1 Introduction

Solar ultraviolet radiation (UVR) exposure is the primary external factor associated with the development of skin cancer and has been classified as carcinogenic to humans (i.e., group 1 carcinogen) by the International Agency for Research on Cancer (IARC) of the World Health Organization (WHO) (1). Solar UVR is the most significant occupational carcinogenic exposure in terms of both incidence and the number of exposed workers (2–4). Outdoor workers (OW) have already been classified as a high-risk occupational category for the development of skin cancer (5). This mostly accounts for keratinocyte carcinoma (KC; more often referred to as non-melanoma skin cancer [NMSC]), which manifests as actinic keratosis (AK, intra-epidermal SCC), invasive cutaneous squamous cell carcinoma (SCC), and/or basal cell carcinoma (BCC) (6–8). SCCs and BCCs account for approximately 99% of all NMSC cases, and one-third of all diagnosed cancer cases annually (9, 10). BCCs are generally associated with intermittent, acute UVR exposure to intermittently sun-exposed skin areas, while SCCs might result from chronic, prolonged UVR exposure. Therefore, SCCs are more prevalent among outdoor workers, as they are exposed to solar UVR for extended periods of time (11).

In 2020, the World Cancer Research Fund classified NMSC as the fifth most common cancer globally, highlighting the urgent need for enhanced reporting and prevention measures, given the high number of unreported cases, with over a million diagnoses and a disregard for risk factors and protective measures. As indicated in the GLOBOCAN 2022 report, there were approximately 20 million new cases of cancer in 2022. Of these cases, over 1.2 million were NMSC (excluding BCC) (12). Despite the relatively low mortality rate associated with this cancer type, particularly when diagnosed in the initial stages, NMSC results in significant morbidity, largely due to the limitations of available treatment options (12, 13). In the present paper, the focus will be on SCC as it is most strongly associated with occupational exposure due to its continuous pattern of exposure to solar UVR.

Occupational safety and health guidelines and laws in many nations still fail to recognize this work-related risk factor, even though millions of workers worldwide are exposed to the occupational carcinogenic exposure represented by solar UVR for a significant portion of their working hours (14). According to a recent German classification for the recognition of UVR-induced occupational diseases, occupational health protection should be provided to any worker who spends more than 50 days outdoors between April and September, between 11 a.m. and 4 p.m. (15). Furthermore, there is no consensus on any specific threshold levels for occupational exposure. The exposure limit recommended by the International Commission on Non-Ionizing Radiation Protection (ICNIRP) of 30 J/m^2^ over an 8-h workday, is based on a biological action spectrum that accounts for both skin and eye effects and is applied to spectral irradiance independently of whether UVR originates from solar or artificial sources (16). A solar exposure corresponding to 30 J/m^2^ when weighted with the ICNIRP action spectrum typically corresponds to approximately 100–130 J/m^2^. An erythemally weighted radiant exposure of 100 J/m^2^ is equivalent to one Standard Erythema Dose (SED) (16). Considering the absence of a specific occupational limit for solar UVR, a recent WHO-ILO working group of experts conducting systematic reviews has used the exposure limit of 1.0–1.3 SED per day (5, 16).

In this context, this paper presents the first Portuguese measurements of personal solar UVR radiation in six outdoor occupations. The findings shall provide better knowledge about occupational solar UVR exposure among outdoor workers and, collaterally, flow into improved occupational safety measures contributing to the protection of outdoor workers from developing occupational skin cancer.

The main objective of this paper was to calculate the excess risk of developing SCC among outdoor workers in Lisbon, based on accurate solar UVR doses measured by digital dosimeters.

## 2 Materials and methods

This study is part of a project entitled “digitally measuring solar ultraviolet radiation in outdoor workers,” also known as the MEAOW project, which has been previously described in detail elsewhere (17). In summary, the objective of the MEAOW study was to increase awareness of solar UVR-induced occupational skin cancers among the public, working population, stakeholders in occupational health and safety, and policymakers in Lisbon and across Portugal. The study was developed on the premise that the relative risk (RR) associated with various patterns of long-term measurements of personal solar UVR exposure differs between subtypes of keratinocyte carcinoma.

### 2.1 Study design

The presented study consists of a modeling study based on the data collected during the MEAOW study—a prospective observational study conducted between the 10th of April and the 31st of October 2023 (7 months) (18). Personal dosimeters were employed, specifically the GENESIS-UV system (19, 20), which enabled decentralized, long-term measurements of solar UVR using data-logging devices.

### 2.2 Setting

The Lisbon Municipality (CML) occupational health services are integrated within the Department of Health, Hygiene and Safety (DSHS), one of the organic units of the Municipal Directorate of Human Resources. DSHS is responsible for promoting policies and implementing actions to promote health and wellbeing at work, as well as ensuring monitoring to create safe places by eliminating/minimizing the risks at CML.

### 2.3 Participants

The MEAOW study (17) included individuals who were engaged in outdoor work activities and employed by the CML. It is important to note that the initial study protocol comprised three occupations: gardeners, masons, and gravediggers (17). However, during the implementation phase and participant recruitment, there was the opportunity to extend to additional outdoor occupations, including gardeners, pavers, gravediggers, asphalters, sanitation workers, and sailors.

### 2.4 Data sources/measurements and variables

The occupational solar UVR exposure monitoring was performed with the GENESIS-UV (GENeration and Extraction System for Individual Exposure) methodology (20, 21). The data collected during the MEAOW study via GENESIS-UV dosimeters provided erythemally weighted irradiance, which is integrated over time (half hour, day) resulting in radiant exposure in joules per square meter (J/m^2^) per half hour and per day (18), i.e., erythemally weighted with the CIE erythemal action spectrum (22). More specifically, the dosimeters were programmed to measure UVA and UVB/C radiation at one-second intervals, with the data weighted using the erythema action spectrum directly on the device through specific filter combinations. Quality control excluded daily records with <5 h of valid measurements or implausible values (e.g., negative readings); dosimeters that failed these criteria systematically were removed from further analyses.

The GENESIS-UV dosimeter’s sensors are routinely verified by the manufacturer to ensure proper calibration. When necessary, recalibration is performed according to national standards. Furthermore, potential deviations caused by aging effects are assessed through internal calibration checks at both the beginning and end of the measurement cycle. These deviations are corrected in the recorded data if required. If the sensor shows a variation exceeding 30% over time, the device undergoes a detailed technical inspection and recalibration by the manufacturer.

During the data collection period, the participants were required to wear the GENESIS-UV personal electronic dosimeter on their left upper arm. Subsequently, the data collected from each dosimeter was transferred to a tablet every Friday by the workers who wore the dosimeter with the help of the supervisor, and initially, from the research team. This data was then transferred to a computer from the GENESIS-UV unit, thereby enabling the automatic reading of data. The experimental campaign took place between spring and fall, i.e., between April (spring) and October (fall) 2023, as previously mentioned.

To ensure adherence to the protocol throughout the seven-month study, several awareness campaigns were conducted at the workplace during breaks, lunch periods, and after work to explain the study’s purpose and invite participation. During this initial implementation phase, the research team anticipated challenges in maintaining worker compliance over the entire period. To mitigate this, dosimeters were rotated within each occupational group during data collection. Each worker wore a dosimeter for 1 week before passing it to a colleague for the following rotation. This process was not entirely linear, as data collection coincided with holiday periods, and participation was voluntary—some workers discontinued wearing the dosimeters after a few weeks. To maximize valid measurements, the research team collaborated closely with on-site supervisors to coordinate rotations and maintain coverage within each occupation. A total of 42 dosimeters were meticulously assembled and subsequently distributed among the six occupational groups participating in the study.

### 2.5 Annual dose estimation

To apply the risk model of Milon et al. (22), which requires annual solar UVR measures, we converted the 7-month MEAOW dataset into full-year estimates. Because observations spanned only 7 months, annual doses were estimated in two steps. First, for each measured month (April–October) we computed the mean daily occupational dose. Mean daily doses can be extracted for each dosimeter, as well as grouped for each occupation. Mean daily doses are thereafter multiplied by the monthly number of working days to obtain monthly doses. The number of working days per month results from the number of working days for a standard Portuguese working year according to Milon et al. (22) with 239 effective workdays (weekends and 22 vacation days excluded). Doses for the unmeasured months (mainly November–March) were extrapolated using relative monthly UV-intensity weighting factors derived from the German ambient UV climatology employed by Wittlich et al. (20). Monthly doses resulting either from measurement data or extrapolation are finally summed up to the annual dose (22). These factors preserved the expected seasonal distribution of solar UVR and allowed the Lisbon measurements to be adjusted to a full-year cycle (22, 23).

### 2.6 Statistical methods

#### 2.6.1 Relative risk (RR) modeling

To estimate the increased risk of an outdoor worker developing SCC, the model developed by Milon et al. (22) was employed. This model/formula expresses the relative risk (RR) as a function of cumulative UVR exposure and age, and it assumes that the person accrues a 25-year history of outdoor work and is 60 years of age. The model developed by Milon et al. (22) assists in estimating the increased likelihood of developing SCC in an individual with a history of outdoor work in comparison to an individual with no such history, by considering the cumulative UV exposure over time. This is expressed by the following formula:


$$\begin{array}{c} RR_{\mathrm{age}=T}=\frac{\mathrm{Ris}k_{B,T}}{\mathrm{Ris}k_{A,T}}={\left(\frac{UV_{\mathrm{tot},B}\;\mathrm{at}\;\mathrm{age}\;T}{UV_{\mathrm{tot},A}\;\mathrm{at}\;\mathrm{age}\;T}\right)}^{\beta }\\ ={\left(\frac{\sum_{Y=T}^{T+25}(UV_{\mathrm{occ}}+UV_{\text{lunch}})+\sum_{T=0}^{60}UV_{\text{recr}}}{\sum_{T=0}^{60}UV_{\text{recr}}}\right)}^{\beta } \end{array}$$

UV_tot_ is the cumulative UV dose received from occupational and recreational exposure. In these formulas, UV_tot,A_ and UV_tot,B_ represent two people of the same age, exposed to two different cumulative doses at an estimated age of 60 years. The model posits that the risk of SCC increases with both age and cumulative UVR exposure and includes an amplification factor *β* = 2.5 derived from epidemiology studies (23).

Our research team considered 25 years of outdoor work to be an unrealistically short exposure period for a 60-year-old individual. Therefore, extra calculations were performed to consider the risk of someone who is 60 years old and has 40 years of outdoor exposure.

#### 2.6.2 Model assumptions and bias

The fundamental premise of the model is that the likelihood of developing SCC increases proportionately with age and the cumulative amount of UVR exposure received during occupational, recreational, and incidental moments (e.g., during lunch breaks). In this model, the fixed value of *β* at 2.5 refers to earlier work by Slaper et al. (23). *β* represents the biological amplification factor for the cumulative UV dose received from occupational and recreational exposure (22). However, the derivation of this specific value remains methodologically ambiguous. Nevertheless, the value was retained in our study for consistency and comparability of findings.

Another assumption of the model pertains to the life-course UVR exposure profile of the two hypothetical comparator individuals: one “outdoor” worker who was exposed to UVR in the course of his/her occupation (including lunch breaks) for a period of 25 years or 40 years, and one “indoor” worker whose exposure was limited to recreational activities throughout his/her until the individual is 60 years old (as the model premise). The total UVR exposure for both indoor or outdoor workers was calculated by combining empirical dosimetry data (for occupational UVR) and literature-based estimates from the Expolis study (for indoor and outdoor recreational UVR) (24), as in the present study, recreational solar UVR was not measured. It is important to note that while occupational doses were measured locally in Lisbon through the MEAOW study using GENESIS-UV personal dosimeters, the recreational exposure values were not directly measured. Instead, they were assumed to be the same as those used in Milon’s study, having been derived from data from Switzerland and Italy (i.e., the recreational dose was derived from Italy) (22). This approach is based on the assumption that there is comparable leisure-time sun behavior and UV intensity across populations, a supposition which may not accurately reflect real-world variations in latitude, cultural habits, standard of living or occupational structure.

Furthermore, the model assumes homogeneity in exposure across individuals in each group; that is to say, all “outdoor workers” and all “indoor workers” have similar patterns of sun exposure within their category. The original formula also assumes that there is a linear accumulation of UVR across 25 years of occupational work and up to 60 years of life. However, it does not consider potential mitigating factors such as the use of sunscreen, protective clothing, or genetic predisposition to skin cancer.

When considered as a whole, these assumptions give rise to potential sources of bias and generalization error, particularly when the model is applied across different populations or geographical areas. However, in the absence of Portuguese-specific baseline SCC risk data and longitudinal clinical outcomes, the Milon model remains a valuable tool for quantifying relative risk from high-quality UVR dosimetry data.

## 3 Results

The dataset for the present study was obtained from the usage of 42 personal electronic dosimeters during 7 months, performing a total of 1,141 days of valid measurements across the six occupations studied (18). The number of dosimeters per occupational group varied depending on the size of the team involved and according to their supervisor’s agreement. In this way, 10 dosimeters were given to two teams of gardeners, 10 were distributed to gravediggers of three different graveyards, eight for one team of asphalters, eight for the one team of sanitation workers, five for one team of pavers, and one for the one sailor.

Ninety individuals, from the above teams, agreed to take part in our study, accepted the invitation letter, and completed the informed consent. The distribution of the outdoor workers per occupation is as follows: there was one Sailor, six Pavers, eight Asphalters, 18 Gardeners, 25 Sanitation workers, and 32 Gravediggers. Pavers, Asphalters, and the Sailor always wore the same dosimeter during the campaign, while in the other occupations, the dosimeters rotated to maintain data collection compliance. For these reasons, the results presented here are based on the dosimeter data collection not directly mean that the data refers to specific individuals.

### 3.1 Average annual occupational UVR dose and SED by occupation

Annual occupational UVR dose and SED calculations were computed by occupational group. The one Sailor had the highest annual occupational exposure to solar UVR (1,087 SED). This one sailor estimates is the same per occupational group and individually. Gardeners, Gravediggers, and Sanitation Workers followed, with similar annual exposures, ranging from 361 to 382 SEDs. Pavers showed the lowest doses of occupational solar UVR (158 SED) (Table 1).

**Table 1:** Relative risk of developing SCC by occupation for 25 and 40 years of outdoor work.

Occupation	Average UV dose [SED]—8 h workday	Relative risk (8 h)	% increased risk (RR-1*100%)
25 years of outdoor occupation			
Sailor	27,163	10.1	912%
Gardeners	9,562	2.9	193%
Gravediggers	9,366	2.9	187%
Sanitation workers	9,031	2.8	179%
Asphalters	7,158	2.3	133%
Pavers	3,951	1.7	65%
40 years of outdoor occupation			
Sailor	43,460	21.9	2,093%
Gardeners	15,299	4.7	371%
Gravediggers	14,986	4.6	360%
Sanitation workers	14,499	4.4	341%
Asphalters	11,453	3.5	246%
Pavers	6,322	2.1	114%

### 3.2 Cumulative UV dose

As the annual recreational UVR dose is considered age-independent, it was assumed to remain constant over the 60-year period for which the relative risk (RR) model is valid (22). Therefore, in terms of recreational UVR dose, a person would receive a total dose of 17,820 SED over a period of 60 years (297 × 60). Additionally, occupational UVR was assumed to remain constant over both 25- and 40-year employment durations (22). This is based on the premise that an individual is employed in the same occupation over the course of these 25 and 40 years and works 7 h per day, plus 1 h for lunch, spent outside. As a result, the cumulative occupational UVR exposure (25 years) was calculated (Table 2). Further assumptions in the case of outdoor workers who have worked outdoors for 40 years and continue to do so were not made.

**Table 2:** UV estimates over 1 year, 25 years, and 40 years of outdoor work.

Occupation	Dosimeter identification	UV dose per year [SED] 239 working days/year	UV dose for 25 years [SED] 239 working days/year	UV dose for 40 years [SED] 239 working days/year
Gravediggers (*n* = 32) 318 days of valid measurements Annual extrapolation of 375 SED	PRT-2023-900-001	515	12,880	20,608
	PRT-2023-900-002	482	12,042	19,268
	PRT-2023-900-003	422	10,562	16,899
	PRT-2023-900-004	263	6,564	10,503
	PRT-2023-900-005	403	10,068	16,109
	PRT-2023-900-006	337	8,414	13,463
	PRT-2023-900-007	484	12,096	19,354
	PRT-2023-900-008	195	4,883	7,813
	PRT-2023-900-009	279	6,981	11,170
	PRT-2023-900-010	367	9,172	14,675
Pavers (*n* = 6) 49 days of valid measurements Annual extrapolation of 158 SED	PRT-2023-900-011	59	1,465	2,344
	PRT-2023-900-014	182	4,546	7,274
	PRT-2023-900-016	93	2,321	3,713
	PRT-2023-900-018	98	2,446	3,913
	PRT-2023-900-019	359	8,979	14,366
Gardeners (*n* = 18) 265 days of valid measurements Annual extrapolation of 382 SED	PRT-2023-900-021	393	9,834	15,735
	PRT-2023-900-022	440	10,992	17,587
	PRT-2023-900-023	339	8,463	13,541
	PRT-2023-900-024	417	10,423	16,677
	PRT-2023-900-025	467	11,675	18,680
	PRT-2023-900-026	219	5,487	8,779
	PRT-2023-900-027	418	10,458	16,734
	PRT-2023-900-028	336	8,400	13,439
	PRT-2023-900-029	286	7,152	11,443
	PRT-2023-900-030	509	12,734	20,374
Sanitation workers (*n* = 25) 352 days of valid measurements Annual extrapolation of 361 SED	PRT-2023-900-031	343	8,576	13,722
	PRT-2023-900-032	430	10,739	17,182
	PRT-2023-900-033	291	7,273	11,637
	PRT-2023-900-034	238	5,953	9,524
	PRT-2023-900-035	331	8,264	13,222
	PRT-2023-900-036	390	9,761	15,617
	PRT-2023-900-037	390	9,756	15,609
	PRT-2023-900-038	477	11,924	19,079
Asphalters (*n* = 8) 150 days of valid measurements Annual extrapolation of 286 SED	PRT-2023-900-039	162	4,038	6,460
	PRT-2023-900-040	177	4,417	7,067
	PRT-2023-900-041	106	2,655	4,248
	PRT-2023-900-042	389	9,714	15,543
	PRT-2023-900-043	287	7,182	11,491
	PRT-2023-900-044	683	17,074	27,318
	PRT-2023-900-045	182	4,551	7,281
	PRT-2023-900-046	305	7,632	12,212
Sailors (*n* = 1) 7 days of valid measurements Annual extrapolation: 1,087 SED	PRT-2023-900-048	1,087	27,163	43,460

### 3.3 Relative risks of developing SCC

Indoor workers are the reference group for the risk estimates, and their occupational exposure was considered to be 0 SEDs, while the same values for recreational exposure to solar UVR were equal for both groups, as referred to in the methods. The RR of developing SCC was calculated by occupational group for 25 and 40 years of outdoor work (Table 1), and the results presented in Table 1 were obtained by the doses presented in Table 2. Obviously, the results from both tables follow the same order of increased risk by occupation, which is also directly related to the measured occupational solar UVR.

Table 3 shows relative risk values for developing SCC per dosimeter, which range from 22% (Paver) to 437% (Asphalter) over 25 years, and from 36 to 921% over 40 years of outdoor work. These minimum and maximum values reflect the range of doses recorded by individual dosimeters and illustrate inter-individual variability within each occupation. This result suggests that working position, working tasks, and their duration can play a role as relevant as latitude and local working conditions (shaded areas). For this reason, the lowest and highest risks per occupation are highlighted in bold in Table 3.

**Table 3:** Relative risk of developing SCC when exposed to solar UVR for a total of 8 h per day (in bold are the highest and lowest risks per occupation).

Occupation	Dosimeter identification	Relative risk for SCC due to 25-year outdoor work	% increased risk (8 h) due to 25-year outdoor work	Relative risk for SCC due to 40-year outdoor work	% increased risk (8 h) due to 40-year outdoor work
Gravedigger (*n* = 32)	PRT-2023-900-001	**3.9**	**290%**	**6.8**	**583%**
	PRT-2023-900-002	3.6	264%	6.2	525%
	PRT-2023-900-003	3.2	220%	5.3	430%
	PRT-2023-900-004	2.2	119%	3.2	218%
	PRT-2023-900-005	3.1	206%	5.0	400%
	PRT-2023-900-006	2.6	163%	4.1	308%
	PRT-2023-900-007	3.7	265%	6.3	529%
	PRT-2023-900-008	**1.8**	**83%**	**2.5**	**148%**
	PRT-2023-900-009	2.3	129%	3.4	238%
	PRT-2023-900-010	2.8	182%	4.5	349%
Paver (*n* = 6)	PRT-2023-900-011	**1.2**	**22%**	**1.4**	**36%**
	PRT-2023-900-014	1.8	76%	2.4	135%
	PRT-2023-900-016	1.4	36%	1.6	61%
	PRT-2023-900-018	1.4	38%	1.6	64%
	PRT-2023-900-019	**2.8**	**177%**	**4.4**	**338%**
Gardener (*n* = 18)	PRT-2023-900-021	3.0	200%	4.9	387%
	PRT-2023-900-022	3.3	232%	5.6	457%
	PRT-2023-900-023	2.6	164%	4.1	311%
	PRT-2023-900-024	3.2	216%	5.2	421%
	PRT-2023-900-025	3.5	252%	6.0	500%
	PRT-2023-900-026	**2.0**	**96%**	**2.7**	**172%**
	PRT-2023-900-027	3.2	217%	5.2	424%
	PRT-2023-900-028	2.6	163%	4.1	308%
	PRT-2023-900-029	2.3	132%	3.5	246%
	PRT-2023-900-030	**3.8**	**285%**	**6.7**	**573%**
Sanitation workers (*n* = 25)	PRT-2023-900-031	2.7	167%	4.2	317%
	PRT-2023-900-032	3.3	225%	5.4	441%
	PRT-2023-900-033	2.4	135%	3.5	251%
	PRT-2023-900-034	**2.1**	**106%**	**2.9**	**192%**
	PRT-2023-900-035	2.6	159%	4.0	301%
	PRT-2023-900-036	3.0	198%	4.8	382%
	PRT-2023-900-037	3.0	198%	4.8	382%
	PRT-2023-900-038	**3.6**	**260%**	**6.2**	**517%**
Asphalters (*n* = 8)	PRT-2023-900-039	1.7	67%	2.2	117%
	PRT-2023-900-040	1.7	74%	2.3	131%
	PRT-2023-900-041	**1.4**	**42%**	**1.7**	**71%**
	PRT-2023-900-042	3.0	197%	4.8	380%
	PRT-2023-900-043	2.3	133%	3.5	247%
	PRT-2023-900-044	**5.4**	**437%**	**10.2**	**921%**
	PRT-2023-900-045	1.8	77%	2.4	135%
	PRT-2023-900-046	2.4	144%	3.7	269%
Sailor (*n* = 1)	PRT-2023-900-048	**10.1**	**912%**	**21.9**	**2,093%**

## 4 Discussion

In the present study, the RR of outdoor workers developing SCC was calculated based on measurements of occupational solar UVR exposure. Higher exposure levels of solar UVR are associated with an increased risk of disease development.

This study provides the first SCC risk estimates for outdoor workers in Portugal using personal electronic dosimeters to assess occupational solar UVR exposure. Occupational exposure to solar UVR increases the risk of developing SCC among outdoor workers, with RR values ranging from 1.2 to 5.4, and from 1.7 for Pavers to 2.9 for Gardeners in terms of occupational groups exposed during 25 years. Higher cumulative occupational UVR exposure over 25 years exhibited a significantly elevated RR for developing SCC. Due to the method for the risk estimation employed and the use of direct occupational measurements, the highest RR (5.4) corresponds to the highest cumulative UV dose of 683 SED per year. Conversely, the lowest RR value (1.2) was associated with a cumulative UV dose of 59 SED over the same period, representing the lowest value.

Risk variability across occupations certainly reflected differences in work conditions and protective behaviors. Gravediggers, Pavers, and Gardeners had higher mean RRs than Sanitation Workers and Asphalters, possibly due to reduced shade access or more time spent outdoors. In contrast, Sanitation Workers often operate from vehicles, which offer UVR protection.

There were also differences between workers of the same group. Lower RR values may be indicative that some outdoor workers mitigate risk through the use of adequate personal protective equipment (PPE). Heightened awareness of environmental potential dangers may encourage outdoor workers to utilize PPE, sunscreen, and seek the shade, thereby reducing the risks associated with prolonged occupational exposure to UVR. It is also worth noting that at CML, certain occupational group supervisors (e.g., Pavers) have the autonomy to choose their work locations, timings of work and breaks, and the ability to choose shaded areas to work. During data collection, it was observed that the pavers supervisor consistently opted for or attempted to work in shade areas when a street needed paving.

Regarding Sailors, work is highly seasonal—mainly from June to September—and often intermittent during voyages. Despite limited continuous exposure, from one worker with 7 days of valid data, he recorded the highest RR, emphasizing that even brief but intense UVR episodes can meaningfully elevate cancer risk. This highlights the importance of considering not just total exposure, but also intensity and context when evaluating occupational UVR risk.

Milon et al. (25) remains the main source of the RR formula used here, though their model was based on estimated, not directly measured, UVR doses. The novel approach of this study is to use actual occupational UVR data from personal dosimeters, as Milon et al. recommended for future research. While several studies have cited Milon’s work, they have predominantly referenced the empirical data without applying the RR formula (26–29). Regarding other studies that attempt to calculate the risk of developing SCC, there is considerable variation between studies. They generally do not employ a RR model like the one employed in this case. Alternatively, they attempt to calculate the risk of developing SCC through systematic reviews and estimations, or qualitative studies (25, 30).

Numerous international studies across diverse geographic and occupational settings consistently demonstrate a significantly elevated risk of SCC among outdoor workers exposed to solar UVR. A European multicenter case–control study reported an odds ratio (OR) of 2.8 for SCC among individuals employed in farming and construction sectors compared to indoor workers (31). National registry data from Bavaria, Germany, found relative risks (RR) of 2.5 in men and 3.6 in women with significant outdoor exposure (32), while the HELIOS-I multicenter study indicated ORs ranging from 2.55 to 2.95 in masons and construction workers (33). The latter finding is corroborated by a German case–control study in 632 incident SCC cases, which shows that SCC-OR doubles at 633 SED lifetime occupational UVR exposure, and then beyond, even rises further exponentially with higher received UVR dosages (34). Furthermore, a global systematic review encompassing data from Europe, Oceania, and the Americas confirmed that 7 of the 7 included studies on SCC observed a significant increase in SCC odds ratio among outdoor workers, varying from 1.4 to 1.9 (6). These findings support the need for effective preventive strategies and recognition of occupational UVR exposure as a carcinogenic risk factor. Only by taking this into account will it be possible to address the global public health challenge of skin cancer and its prevention effectively in the future.

### 4.1 Generalizability of the study results

When extrapolating the findings, it is important to acknowledge that the measurement campaign was conducted during the months of the year with the higher daily radiant exposure, which may lead to an overestimation of exposure levels when generalized to other periods of the year. Daily radiant exposure if influenced by seasonal variations in daylight duration. In addition, not all individuals engaged in the outdoor activities studied have identical working hours and tasks. For example, sanitation workers in are routinely engaged in night shifts, experiencing minimal exposure to sunlight during their assigned work schedules. This might not apply to all Lisbon councils, therefore, to ensure the accuracy of extrapolating the RR estimates for the wider Lisbon district, it would be prudent to replicate the MEAOW study methodology with a larger and more geographically diverse cohort of outdoor workers.

Portugal’s geography and climate result in considerable variability in UVR intensity, which is commonly expressed using the UV Index, a standardized indicator of the potential for erythema caused by solar UV radiation (35). Higher-altitude inland areas may experience greater UVR intensity, while northern coastal areas are generally cloudier and therefore exhibit lower UVR intensity. In contrast, southern regions such as the Algarve, central mountainous zones, and eastern inland areas (around Portalegre), frequently exhibit higher UV Index values, particularly during the summer months when cloudiness is lower (36, 37). Moreover, the islands of Madeira, due to their lower latitudes, can also experience higher UVR intensity, and the Azores islands’ latitudes are frequently covered by orographic clouds and/or large-scale cloudiness, which generally limits UVR intensity. Even though the RR estimates are based on UVR data from Lisbon, the country’s Mediterranean climate suggests high cumulative exposures in other regions. These differences are also reflected in terms of occupational groups, varying substantially in accordance with the geographical region. Agriculture predominates in rural regions such as Alentejo and the Douro Valley, while fishing and maritime activities are more prevalent in the Algarve and the Azores. These regional differences may shape UVR exposure and influence the risk of developing SCC, reinforcing the need to replicate this study across diverse geographical and occupational contexts.

### 4.2 Limitations

The recreational UVR values employed in the RR formula were sourced from Milon et al., based on indoor workers in Milan (45°N) (22), and it may not describe the recreational UVR exposure of the outdoor workers in Portugal. The city of Lisbon, located at a latitude of 38°N, is likely to experience higher levels of UVR due to its lower latitude (38) and milder climate during the winter, which is therefore more suitable for outdoor activities. This may have resulted in an underestimation of the risk of SCC in our analysis. While local atmospheric conditions, such as cloud cover, may potentially influence these findings, the absence of location-specific recreational UVR data remains a significant limitation. It is recommended that future research incorporate region-specific UV Index and environmental data. This would allow for the refinement of exposure estimates and improvement in the precision of the models. These findings would also be strengthened by the inclusion of statistical measures such as confidence intervals or *p*-values to indicate precision and significance. Future analyses should include at least confidence intervals around RR estimates to enhance the interpretability and robustness of the results, particularly when comparing occupational groups or when drawing inferences for policy recommendations.

The use of personal electronic dosimeter on the left “upper arm” is also a limitation of the study as there are differences in radiant exposure along the body. The study sample size was relatively small, and certain occupational categories, such as Sailors, were underrepresented, with only one dosimeter collecting data over a short period. This limited representation and may compromise the reliability of the RR values calculated for such groups.

### 4.3 Policy and public health implications

These findings have important implications for occupational health policy and the protection of outdoor workers. Given the elevated risk of SCC linked to long-term UVR exposure, there is a compelling case for the formal recognition of SCC as an occupational disease. At a global and national level, health policies may formalize evidence-based, comprehensive protection and prevention strategies for outdoor workers, critically supported by accurate, measured personal UVR dosimetry data. The direct measurement approach, by using actual occupational UVR data from personal electronic dosimeters to calculate SCC risk estimates, provides more reliable data for risk assessment than other data used from different geographical regions and climates. The capability of these dosimeters for decentralized, long-term measurements is crucial for understanding exposure patterns and informing policy formulation, particularly given the significant underreporting and the varying, yet consistently high, exposure levels across diverse outdoor occupations.

At a national level, employers should implement protective measures according to the hierarchy of controls in risk management and TOP principles (technical, organizational, and personal measures), such as providing shades (technical measures), schedule breaks and regular skin checks for early detection (organizational measures), and UV-protecting clothing (personal measures). Furthermore, routine UVR monitoring could be part of occupational health surveillance in high-exposure professions, enhancing early intervention and prevention efforts. To ensure that similar contexts have the necessary legislative, administrative, and technical capabilities to implement occupational UVR policies effectively, capacity-building actions must be bolstered. This will contribute to a global, harmonized approach to prevent SCC. Nevertheless, these results have already been presented to the Lisbon Municipality health department.

## 5 Conclusion

This study presents the first SCC risk estimates for Portuguese (Lisbon) outdoor workers based on solar UVR exposure measurements by personal electronic dosimeters. Such data can improve the current understanding of the health risks associated with solar UVR exposure and the prevention of NMSC, more specifically SCC, among individuals engaged in outdoor work. The findings indicate an elevated SCC risk among these individuals, reinforce the value of direct dosimetry for accurate risk assessment, and highlight the necessity of comprehensive protective strategies. Moreover, they contribute to the advancement of public health strategies in Portugal, in the domains of epidemiology, public health, and occupational health and safety, as well as clinical research.

## Data Availability

The raw data supporting the conclusions of this article will be made available by the authors without undue reservation.
